# A glimpse into the future: Integrating artificial intelligence for precision HER2‐positive breast cancer management

**DOI:** 10.1002/imo2.19

**Published:** 2024-08-02

**Authors:** Xinpei Deng, Yixuan Yan, Zekai Zhan, Jindong Xie, Hailin Tang, Yutian Zou, Jian Tu, Peng Liu

**Affiliations:** ^1^ State Key Laboratory of Oncology in South China, Guangdong Provincial Clinical Research Center for Cancer Sun Yat‐sen University Cancer Center Guangzhou China; ^2^ Guanghua School of Stomatology Sun Yat‐sen University Guangzhou China; ^3^ School of Medicine Sun Yat‐sen University Shenzhen China; ^4^ Pharmacy School of Guilin Medical University Guilin Guangxi China

**Keywords:** anti‐HER2 therapy, artificial intelligence, breast cancer, image recognition, radiogenomics

## Abstract

Breast cancer (BC), specifically HER2‐positives subtype, has a poor prognosis. Nevertheless, the development of anti‐HER2 therapy yielded satisfactory outcomes. Therefore, evaluating patient HER2 status and ascertaining responsiveness to anti‐HER2 therapy is crucial. The advent of deep learning has propelled the artificial intelligence (AI) revolution, leading to an increased applicability of AI in predictive models. In the field of medicine, AI is an emerging modality that is gaining momentum for facilitating cancer diagnosis and treatment, particularly in the effective management of breast cancer. This study aims to provide a comprehensive review of current diagnostic and predictive models that utilize data obtained from histopathological slides, radiomics, and HER2 binding sites. Advancements and practical applications of these models were also evaluated. Additionally, we examined existing obstacles that AI encounters for anti‐HER2 therapy. We also proposed future directions for integrating AI in assessing and managing anti‐HER2 therapy. The findings of this study offer valuable insights into the evaluation of AI‐based anti‐HER2 therapy, emphasizing key concepts and obstacles that, if addressed, could facilitate the integration of AI‐assisted anti‐HER2 therapy. The integration of AI has the potential to enhance the precision and customization of screening and treatment protocols for HER2+ breast cancer.

## Highlights

- We first reviewed the application of AI in assisting HER2 status evaluation and anti‐HER2 therapy.
- Research progress and applications of the current diagnostic and prediction models based on pathology and radiology have been reviewed.
- Then, we also discuss the current dilemmas encountered by artificial intelligence (AI) in the field of anti‐HER2 and the future directions that need to be headed.

## Graphical abstract

This review provides a systematic overview of existing AI studies related to anti‐HER2 therapies in breast cancer (BC). It delves into three key domains of artificial intelligence (AI) research associated with BC anti‐HER2 therapies, namely the prediction of HER2 status, the provision of assistance in anti‐HER2 therapy, and the prospective directions for the advancement of AI studies concerning HER2‐positive BC.

## 1 INTRODUCTION

Breast cancer (BC) is one of the most common diseases among women [1]. Using immunohistochemistry (IHC) parameters, BC can be classified into four subtypes, with HER2‐positive breast cancer (HER2+ BC) accounting for 15%–20% of all cases [2]. HER2 subtype has poor prognosis [3]. The HER2 oncogene encodes a transmembrane receptor tyrosine kinase that is a member of the epidermal growth factor receptor (EGFR) family [4]. The activation of downstream signaling pathways (mainly PI3K/AKT/mTOR and Ras/Raf/MEK/MAPK) stimulates cell proliferation, survival, and angiogenesis [5]. Because of its indispensable role in BC progression, HER2 is considered an effective target for HER2+ BC. Generally, anti‐HER2 therapies are divided into four categories: therapeutic antibodies, antibody‐drug conjugates (ADCs), tyrosine kinase inhibitors (TKIs), and anti‐HER2 combination therapies [6]. In 1998, the Food and Drug Administration (FDA) approved trastuzumab as the most iconic therapeutic antibody. Subsequently, numerous novel antibodies, such as Pertuzumab and Margetuximab, have been discovered. These antibodies exhibit either enhanced specificity in binding to the HER2 receptor compared to trastuzumab, or they possess the ability to bind to additional epitopes, thereby augmenting their activity and eliciting heightened immunologic responses. Recently, novel pharmaceutical agents, including ADCs and TKIs, have been developed for the targeted delivery of cytotoxic drugs to tumors and the inhibition of tyrosine kinases, respectively. Although numerous agents are valuable, some patients may not respond or become resistant to them. Therefore, the development of new targeted agents is essential. Preclinical and clinical trials involving PI3K/AKT/mTOR inhibitors, PD‐1/PD‐L1 inhibitors, HER2‐targeted vaccines, and CAR T‐cell therapy have offered promising anti‐HER2 combined strategies [6]. As the preferred regimen for HER2+ BC, Trastuzumab with Pertuzumab and Taxane remain the gold standard [7].

## 2 HARNESSING THE POWER OF ARTIFICIAL INTELLIGENCE IN BREAST CANCER TREATMENT

McCarthy et al. [8] coined the term artificial intelligence (AI) in the 1950s to describe the field of computer science where machine‐based approaches are utilized to make predictions, mimicking intelligent human actions in similar situations. Simultaneously, clinical oncology generates “big” data, whether from molecular, imaging, pharmacological, or biological sources. This presents clinicians and data scientists with various challenges in analyzing the data while contributing to the discovery of cancer biology and treatment [9]. Currently, AI‐driven advancements in cancer diagnosis have undergone continuous refinement, with many achieving or surpassing human diagnostic accuracy [10, 11]. AI approaches, including diagnosis, prediction of treatment responses, and identification of treatment‐related changes, are increasingly assisting in complex decision‐making tasks. Furthermore, discoveries have been made in imaging representations of phenotypic characteristics and genotypic features associated with prognosis.

Specifically, AI has shown great potential for treating BC. The CSCO Breast Cancer Diagnosis and Treatment Guidelines 2022 mentioned that experts should encourage clinical research related to AI and developing AI systems with intellectual property rights. In early 2010, computer‐aided detection (CAD) was used for screening and diagnostic mammography. Lee et al. [12] applied machine learning to predict prognostic biomarkers and molecular subtypes of BC based on tumor heterogeneity and angiogenesis observed on magnetic resonance imaging (MRI). AI was successfully applied to BC detection, paving the way for further applications in a wider range of diagnostic tasks, including tumor classification and cancer detection. Wang et al. [13] enhanced BC classification through integrating graph convolutional network with convolutional neural network. With AI, images of pathologically stained slides (HE staining, IHC staining, etc.) can be processed using an image processing model (usually a machine learning approach), which can directly decipher hormone and HER2 receptor status phenotypes [14]. Taleghamar et al. [15] investigated the efficacy of multiparametric quantitative ultrasound imaging using unsupervised classification algorithms. Malignancy prediction with cone‐beam CT was better with machine learning techniques than with humans in terms of AUC and specificity. Uhlig et al. [16] conducted an assessment of the diagnostic efficacy of machine learning algorithms for predicting malignancy utilizing breast cone‐beam CT (CBCT), revealing that they outperformed human readers in both AUC and specificity. In a similar study, Mazurowski et al. [17] extracted an array of imaging features from DCE‐MRI scans to predict distant survival in patients with BC using computer vision.

**Figure 1:**
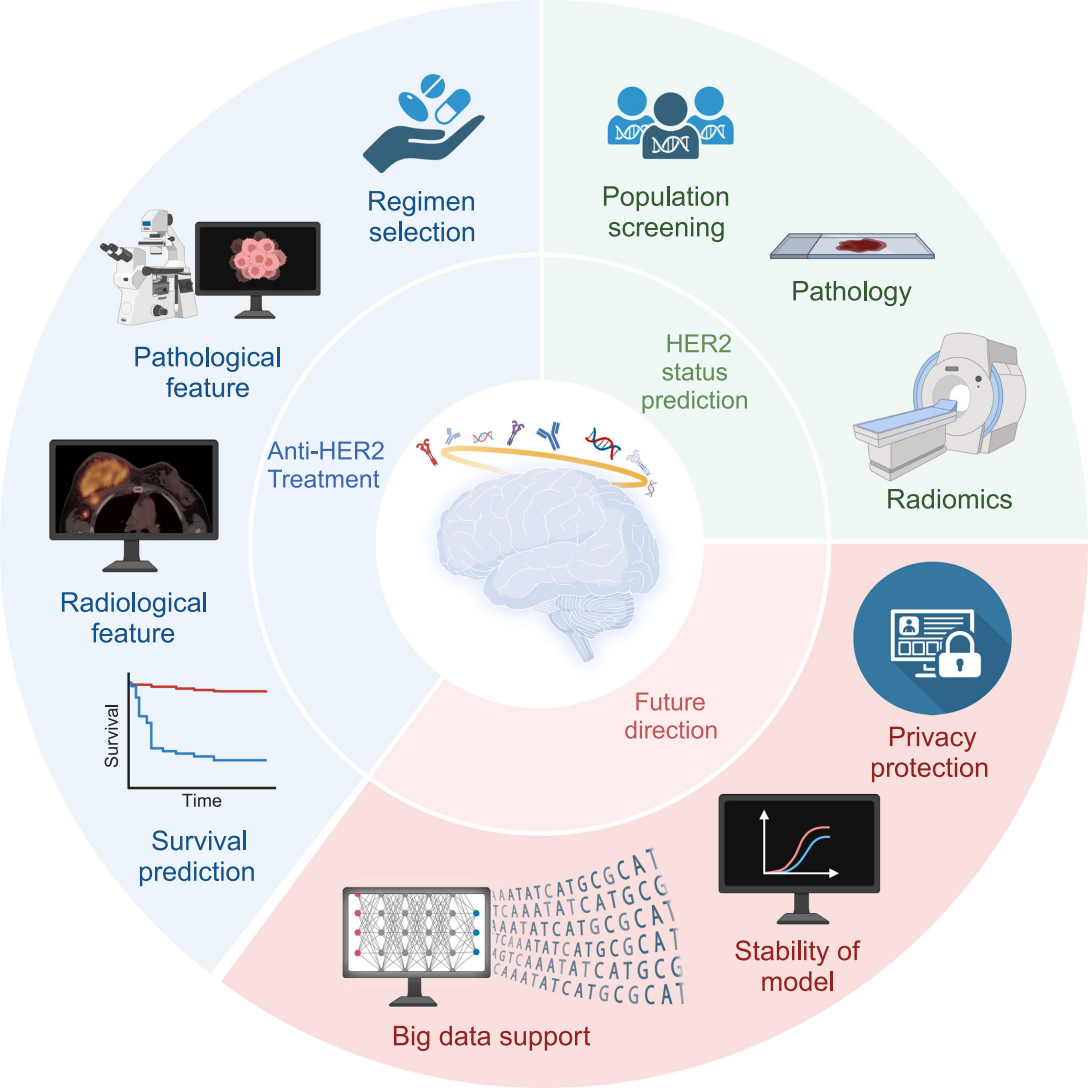
This review provides a systematic overview of existing AI studies related to anti‐HER2 therapies in BC. It delves into three key domains of AI research associated with BC anti‐HER2 therapies, namely the prediction of HER2 status, the provision of assistance in anti‐HER2 therapy, and the prospective directions for the advancement of AI studies concerning HER2‐positive BC. AI, artificial intelligence; BC, breast cancer.

This review provides a systematic overview of existing AI studies related to BC anti‐HER2 therapies in three areas: HER2 status prediction, anti‐HER2 therapy assistance, and the future developmental direction of AI studies related to HER2+ BC (Figure 1).

## 3 AI IN EVALUATING HER2 STATUS

**Figure 2:**
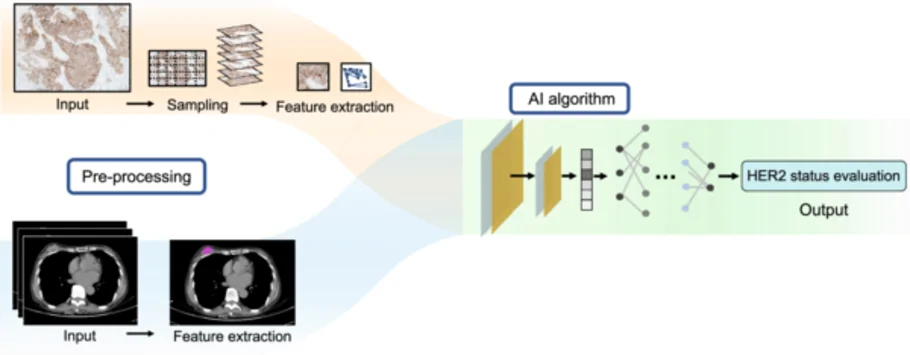
AI‐based prediction of breast cancer HER2 status. AI‐based prediction of breast cancer HER2 status can generally be divided into three steps: preprocessing, AI algorithm, and HER2 status output. In the preprocessing step, raw data, such as tissue images or radiological images, undergoes preprocessing to eliminate irrelevant information. This step is crucial to enable the AI algorithm to learn from the data and generate accurate predictions. In the AI algorithm step, various AI algorithms, including deep learning and machine learning, may be employed to predict HER2 status. In the HER2 status output step, AI aids clinicians in evaluating HER2 status and planning the subsequent course of treatment.

Accurate evaluation of HER2 status is essential for precision therapy. Therefore, HER2 status evaluation is becoming increasingly complex and demanding for healthcare providers. Pathological and radiological examinations are the most widely used methods for classifying HER2 status. AI plays a crucial role in these fields and their applications are described in detail in the following sections (Figure 2).

### Pathological examination

**Figure 3:**
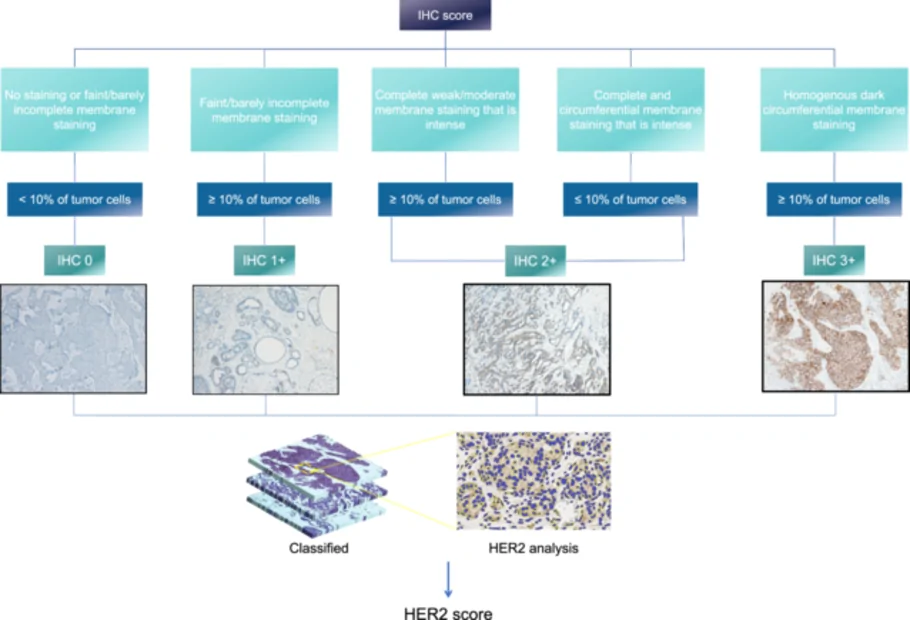
AI assists HER2 score in pathology. The revised scoring criteria for breast HER2 IHC testing, as published by ASCO/CAP, are depicted in the figure. ASCO/CAP, American Society of Clinical Oncology/College of American Pathologists; IHC, immunohistochemistry.

Despite significant advances in alternative therapeutic approaches such as serum marker detection, pathology remains the gold standard for cancer diagnosis. IHC is one of the most widely used pathological methods for evaluating the HER2 status. Figure 3 displays the scoring criteria for breast HER2 IHC testing revised by the American Society of Clinical Oncology/College of American Pathologists (ASCO/CAP) in 2018 and how AI assists with HER2 score [18]. In the HER2‐level scoring system, scores of 0 and 1+ indicate HER2 negativity, while a score of 3+ indicates HER2 positivity. Cases scoring 2+ are considered equivocal and require follow‐up with in situ hybridization (ISH) [18]. The latest literature proposes a potential new nomenclature for those with a HER2 IHC score of 1+ or 2+ and a negative ISH assay, namely HER2 low BC [19]. Precisely classifying patients with different HER2 statuses is crucial; however, histological interpretation of biopsy results by pathologists is subjective and semiquantitative, leading to large interobserver and intraobserver variations [20]. Additionally, manual image analysis requires time and effort and is error‐prone, which may affect treatment selection. As quantitative analysis of pathological images becomes increasingly important, studies concentrated on automated analysis.

Several image analysis techniques were designed to automate repetitive tasks in digital pathology, where the human‐determined truth is clearly evaluated by a human expert. These techniques improved interobserver agreement [21, 22], but still require some level of user intervention. Recently, deep learning (DL), a burgeoning AI technology, has revolutionized image‐recognition approach [23]. This made it possible to achieve fully automated and accurate computer‐aided diagnoses. DL in pathological image analysis can be roughly divided into two steps: preprocessing and AI algorithms [24]. Figure 3 shows the typical steps for HER2 score criteria and histopathological image analysis with AI.

One challenge in preprocessing whole slide images (WSIs) is their very large size. WSIs can contain tens of billions of pixels, which increases the number of parameters to be estimated as well as the computational power and memory required. One approach for this challenge is to resize the image. Wang et al. [25] resized WSIs to a smaller one, which improved the performance of the proposed network. However, image resizing loses information at the cellular level. Some HER2 automated scoring studies divided WSI into partial regions [26]. Since entire WSIs can contain hundreds of thousands of patches, false positives will likely appear. Khameneh et al. [27] used a superpixel‐based support vector machine in conjunction with a feature‐learning classifier to differentiate between epithelial and stromal regions within WSIs. Their approach improved the efficiency of the classification task and reduced the number of input variables. Most research methods for HER2 score classification rely heavily on large data sets, are computationally expensive, and are ineffective in improving HER2 score recognition. Ordinary neural networks used for image recognition and processing can be used to automatically extract features from tissue samples and can be trained on large data sets. The width of the neural network refers to the number of neurons in a layer. A wider network has more neurons in each layer, which helps it learn more complex data patterns. However, wider networks can also be more computationally expensive to train and require more data to generalize effectively. Zheng et al. [28] designed a neural network with an extended width for these issues. They proposed a parallel‐wide separable (WP) structure with convolution kernels of varying sizes that improved the recognition rate and reduced calculation cost. The AI algorithm, after preprocessing, is a key step in histopathological image analysis. Convolutional neural networks (CNNs) are the most researched DL models for medical image understanding tasks. Table 1 shows the different algorithms for automated HER2 scoring and their performances. HER2 status can be determined by either assessing the expression of HER2 protein or measuring ERBB2 gene amplification on chromosome 17 using ISH [18]. Fluorescence signals often form clusters where single signals cannot be identified unambiguously. Pathologists are advised to estimate the number of signals in clusters based on their experience [29]. Nevertheless, such estimations are error‐prone and subjective. Several studies have attempted to identify effective automated methods for FISH signal analysis. Binder et al. [30] proposed an algorithm designed for the purpose of global spot counting in stacked three‐dimensional slice FISH images, specifically capable of functioning effectively in the presence of complex backgrounds. The algorithm predicted HER2 status with an accuracy of 92.6%. Xue et al. [31] presented a deep‐learning‐based method for the automated detection of HER2 amplification status in FISH images. This method consists of two steps: a tumor cell nucleus detector and a signal detector. The tumor cell nucleus detector used the You Only Looking Once version 3 (YOLOv3) architecture to localize the tumor cell nuclei in a FISH image. The signal detector then employs a deep convolutional neural network (DCNN) to classify the nuclei as either HER2‐positive or HER2‐negative. This method was evaluated using a data set of 918 FISH images obtained from 320 consecutive cases of invasive BC. The method demonstrated an overall classification accuracy of 85.33% (157 out of 184) with a mean average precision of 0.735. Palm and colleagues have shown that a combined HER2 IHC and ISH AI workflow is possible, achieving a Cohen's *κ* of 0.94 when evaluated following the ASCO/CAP guidelines [32]. Several contests were conducted to compare and advance state‐of‐the‐art AI‐based automated methods for HER2 scoring. In Qaiser's study [33], the contest data set comprised digitized WSIs of sections from 86 cases of invasive breast carcinoma stained with IHC for HER2. Participants predicted scores on the IHC slides, which were then compared with the ground truth (a consensus score from at least two experts). They also organized a man‐versus‐machine event where one of the automated methods slightly surpassed the performance of the top‐performing participant pathologist. Recent advancements in the field of anti‐HER2 ADCs have presented novel therapeutic possibilities for breast cancer treatment. Recent clinical trials focusing on metastatic breast cancers have demonstrated the efficacy of Trastuzumab Deruxtecan (T‐Dxd), an anti‐HER2 ADC, in both HER2‐positive breast cancers and those exhibiting HER2‐low expression [34, 35]. The differentiation between IHC‐0 and IHC1+, previously considered clinically insignificant, has now garnered significant attention, resulting in the reclassification of HER2‐negative cancers into HER2‐low and HER2‐zero subtypes [34, 35, 36]. Owing to the growing attention and heightened focus on the HER2‐low subgroup, Wu et al. [37] devised an AI algorithm to enhance the precision and uniformity of the evaluation process for HER2 IHC 0 and 1+. By incorporating AI assistance, the accuracy and consistency of HER2 IHC 0 and 1+ assessment, as well as evaluating HER2 IHC in BC with heterogeneity, can be substantially enhanced through AI‐assisted interpretation.

**Table 1:** Applying deep learning to predict HER2 status.

References	Data set	Base	Remarks	Year
Saha and Chakraborty [26]	752 core images cropped from 79 WSIs	Fully connected LSTM recurrent network, cell membrane and nuclei detection	98.33% accuracy	2018
Wu et al. [37]	246 IDC HER2 IHC	Heatmap regression using fully convolutional networks in an end‐to‐end manner	HER2 0 cases 93% accuracy HER2 1+ cases 89% accuracy	2023
Wang et al. [25]	334 WSIs	Residual network	93% accuracy	2022
Zheng et al. [28]	2060 images	WP structure	95.75% accuracy	2022
Vandenberghe et al. [38]	74 WSIs	Color deconvolution, watershed segmentation, support vector machine, random forest, CNN, HER2 scoring by classifying cells	83% accuracy	2017
Qaiser and Rajput [39]	86 WSIs	Data augmentation, deep reinforcement learning, ROI‐based score prediction	79.4% accuracy	2019
Singh and Mukundan [40]	1345 core regions from 52 WSIs	Intensity and color features, neural network classifier	91.1% accuracy	2018
Cordeiro et al. [41]	2580 patches from 86 WSIs	support vector machine classifier	90% accuracy	2018
Khameneh et al. [27]	127 WSIs	superpixel‐based SVM classifier, CNNnan	87% classification accuracy	2019

Abbreviations: CNN, convolutional neural network; IDC, invasive ductal carcinoma; IHC, immunohistochemistry; LSTM, long short‐term memory; ROI, region of interest; SVM, support vector machine; WP, wide pyramid; WSI, whole slide images.

The accuracy of HER2 diagnosis was improved by several automated scoring methods using AI models. Using computer technology to analyze these samples would provide important scientific and medical information, as well as automate routine diagnostic tasks [42]. However, these methods remain at the research level rather than being used in routine clinical care. One reason for this is that interpreting pathology slides is still considered a subjective process. There is a concern that automated scoring methods may not fully replicate the expertise of human pathologists. Another reason is that the cost and ethical issues associated with annotating large datasets of pathology slides have not yet been fully resolved.

### HER‐2 status evaluation in radiomics signatures based on images

Conventional HER2 assessment using needle biopsy samples may not capture all tumor features. This is due to the small size of the tissue samples and tumor heterogeneity, leading to false‐negative biopsy results for individual tissues. Radiomics is a high‐throughput technique used to extract quantitative features from medical images. Recently, radiomics was used to extract many nonvisible imaging features from X‐ray, ultrasound (US), MRI, and positron emission tomography‐computed tomography (PET‐CT) images. DL has led to a surge radiomics because DL can be used to extract complex features from medical images that cannot be extracted using traditional methods [23].

Radiomics employs diverse mathematical and statistical techniques to extract features like shape, intensity, and texture from medical images. Several studies have investigated using AI models to classify BC subtypes using radiomic signatures. Wu et al. [37] extracted radiomics features from ultrasound images to predict the expression of molecular biomarkers. The results indicated that the models achieved a predictive performance with an AUC of 0.74. Li et al. [43] identified 25 high‐throughput features for HER2 (AUC = 0.730) that closely correlated with HER2 biological properties. Park et al. [44] evaluated the performance of four popular machine learning models using low‐dose perfusion computed tomography (CT) to predict prognostic biomarkers and molecular subtypes. Their results showed that the random forest model achieved excellent results with an accuracy of 83% and an AUC of 0.88 in predicting HER2 overexpression. Ha et al. [45] predicted BC molecular subtypes using MRI with CNN algorithm. The CNN algorithm used in their study achieved an overall accuracy of 70% with an AUC of 0.888 for the HER2 subtype. Demircioglu et al. [46] presented a model with an AUC of 0.75 for predicting HER2‐enriched molecular subtype.

Although AI models can predict the molecular subtypes of BC, large and diverse data sets are required to improve their predictive performance.

## 4 AI FOR ASSISTING ANTI‐HER2 TREATMENT

Chemotherapy combined with anti‐HER2 agents is the standard of care for patients with HER2+ BC and is referred to as neoadjuvant chemotherapy [47]. There are numerous methods for evaluating the therapeutic effects of anti‐HER2 agents, among which complete pathological response (pCR) represents a particularly important assessment criterion. Patients with pCR are more likely to have a favorable outcome than those with partial or no response [48]. Nevertheless, not all patients with clinically defined HER2+ breast cancer respond to neoadjuvant chemotherapy (NAC). Among HER2+ cases, the response as well as overall survival rates of NAC range from 10% to 41% and 56%–85%, respectively [49, 50, 51, 52, 53, 54, 55, 56]. Furthermore, while combining trastuzumab and chemotherapy is effective for treating breast cancer with HER2 overexpression, it is associated with adverse effects and irreversible drug resistance, which can negatively impact patient quality of life [57]. Studies have focused on improving the efficacy of targeted therapy for HER2+ BC.

Predicting treatment response in advance can help avoid unnecessary treatment costs and enable the design of a more suitable and precise therapeutic schedule, ultimately reducing treatment duration. To prevent over‐ or under‐treatment of HER2+ BC, there is an urgent need for a predictive model. Moreover, as one of the most malignant subtypes of breast cancer, HER2+ cases have serious complications such as the highest incidence of brain metastases [58]. By predicting the likelihood of brain metastasis at an earlier stage, patients can receive treatment sooner to prevent neurocognitive decline and improve their quality of life.

**Figure 4:**
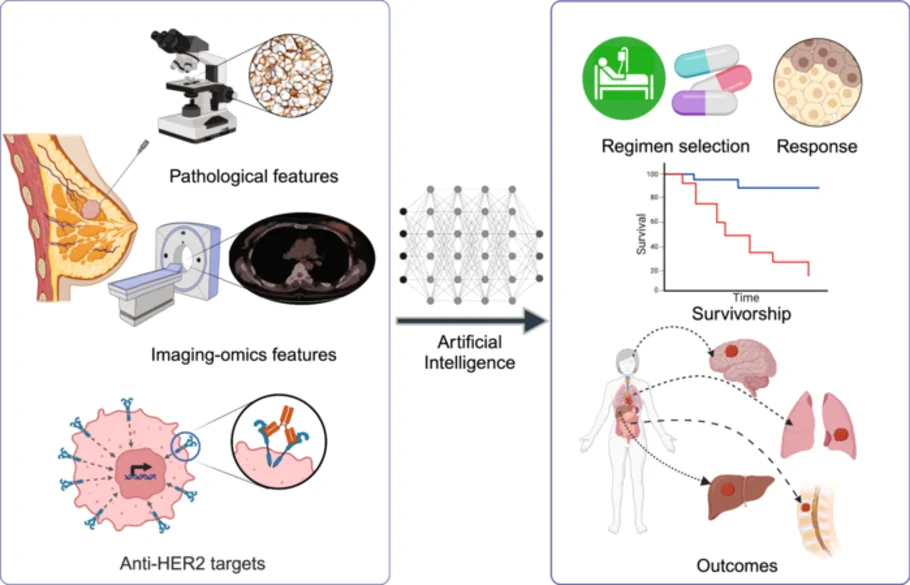
Applying artificial intelligence to breast cancer anti‐HER2 therapy. AI holds promise in transforming the management of BC through the provision of individualized assessments of patient reactions to anti‐HER2 therapy. AI‐driven technologies can be employed to evaluate diverse datasets encompassing pathological characteristics, imaging‐omics attributes, and anti‐HER2 markers, thereby facilitating the anticipation of treatment regimens, treatment response, patient survival, and overall prognosis.

In this section, we introduce the application of AI in assisting anti‐HER2 therapy in the following four aspects in detail, as shown in Table 2 and Figure 4.

**Table 2:** AI‐based approaches in assisting anti‐HER2 therapy.

Reference	Data set	Model/algorithm	Purpose	Remarks
Farahmand et al. [48]	85 WSIs	CNN	Predicting response to NAT	Annotated model: AUC = 0.80 (slide‐level)
Bychkov et al. [59]	712 WSIs	CNN	Predicting DDFS	AUC = 0.67
Yang et al. [60]	127 WSIs, clinical data	CNN	Predicting recurrence	AUC = 0.72
Cain et al. [61]	288 DCE‐MRI images	Machine learning	Predicting response to NAT	AUC = 0.707
Bitencourt et al. [62]	311 MRI images	Machine learning	Predicting response to NAT	86.5% sensitivity, 80.0% specificity, 83.9% accuracy
Mazurowski et al. [17]	892 DCE‐MRI images	Computer vision algorithms	Identifying prognostic features of DFS	/
Wu et al. [63]	246 perfusion‐MRI images	Network analysis	Predicting recurrence	HR: 3.42 (1.55–7.57)
Drukker et al. [64]	157 MRI images	RNN	Predicting 2‐year RFS	AUC = 0.80
Yu et al. [65]	Multiomic signatures from 1088 patients	SVM	Predicting ALN status	AUC = 0.93 (prospective‐retrospective validation cohort)
Dashevsky et al. [66]	109 MRI images	SVM	Predicting negative surgical margins	74.4% specificity, 71.4% sensitivity
Jiang et al. [67]	592 US images	DL	Predicting response to NAT	AUC = 0.95
Liu et al. [68]	393 longitudinal US images	Siamese multitask network	Predicting response to NAT	AUC = 0.902, 0.957
Saini and Agarwal [69]	519 compounds inhibitory to EGFR	Knowledge‐based computational classification model	Identifying HER2‐specific inhibitors	AUC = 0.95
Balakrishnan et al. [70]	193 mutants of trastuzumab	SVR	Identifying affinity improved trastuzumab	$R^{2}\mathrm{pre}=0.72$
Takada et al. [4]	776 HER2+ patients	Machine learning	Predict brain metastasis	AUC = 0.871

Abbreviations: ALN, axillary lymph nodes; AUC, area under curve; DCE‐MRI, dynamic contrast‐enhanced magnetic resonance imaging; DDFS, distant disease‐free survival; DFS, disease‐free survival; DL, deep learning; EGFR, epidermal growth factor receptor; HR, hazard ratio; MRI, magnetic resonance imaging; NAT, neoadjuvant therapy; RFS, relapse‐free survival; RNN, recurrent neural network; SVR, support vector regression; US, ultrasound.

### AI predicts anti‐HER2 therapy efficacy by pathological features

#### Response prediction and prognosis forecast

One of the most widely used methodologies in pathology is hematoxylin and eosin (HE) staining. The morphological characteristics of the nucleus in HE‐stained images play a crucial role in predicting the prognosis of various malignant tumors [71]. Additionally, the abundant information contained in these images can be used to track disease progression, predict survival, and design personalized treatment options. However, traditional histopathological techniques may not meet the demands of precision medicine owing to the significant workload required for experts to extract information from complex images [72]. Currently, using computer‐aided predictors for WSIs is becoming increasingly popular. This is because, aside from their ability to analyze pathological characteristics, they have the advantages of radiomics, such as low cost, high speed, and noninvasiveness.

AI‐driven digital pathology has demonstrated efficacy in tumor diagnosis and treatment, showcasing significant potential in enhancing the precision of pathological assessments, devising treatment strategies, forecasting patient prognoses, and alleviating manual labor [73]. Two computational methods are commonly used to analyze pathological images: conventional machine learning and DL [74]. CNNs are predominant DL models for pathological image analysis [23]. They can be used for tumor detection, quantification of cell characteristics, and classification of small tissue images [75]. Abdel‐Zaher et al. [76] and Zhi et al. [77] trained deep belief networks and transfer learning, respectively, to diagnose BC. Kather et al. [78] developed a deep residual learning model that could forecast microsatellite instability (MSI). Compared with non‐AI methods, CNN models demonstrated superior accuracy. Hence, AI overcomes subjective bias by directly processing biomedical images and extracting features from histological images.

Currently, there is a strong correlation between HER2+ BC morphology and the efficacy of anti‐HER2 targeted therapies. This allows for improved prediction of treatment outcomes. In pathology, a reliable pattern has emerged for predicting treatment efficacy; to predict response, HER2 status is checked first. Farahmand et al. provided a HE‐based algorithm that can predict trastuzumab response in HER2+ BC [48]. A novel CNN approach was developed to predict the treatment outcomes. Using WSIs from 85 HER2+ patients who received treatment, the classifier was trained to achieve an AUC of 0.80 in predicting pCR. Manual annotation of regions of interest at the tile level significantly improved the prediction accuracy for both HER2 status and trastuzumab response. This suggests that incorporating human supervision into AI can improve its performance. Accurate prediction can facilitate faster and more cost‐effective treatment selection. HER2 amplification detected by ISH or excess HER2 protein detected by IHC is commonly used to identify patients who may benefit from anti‐HER2 agents. These standards are widely accepted. However, some borderline cases challenge this classification, suggesting that modifications may be necessary [18]. Farahmand and colleagues reported that not all clinically defined HER2+ patients respond to neoadjuvant chemotherapy. Conversely, tumors lacking HER2 amplification may benefit from anti‐HER2 targeted therapy [48]. Bychkov et al. [59] developed a CNN model weakly supervised by HER2 gene proliferation conditions using digital HE‐stained images. The CNN‐predicted morphology‐based HER2 status was referred to as the HE‐HER2 score. Here, the association between HE‐HER2 scores and distant disease‐free survival (DDFS) was assessed. Patients with CISH HER2+ BC treated with trastuzumab who had a machine learning‐derived HE‐HER2 score higher than the median had a better DDFS. These findings demonstrate that HER2‐related morphology is associated with anti‐HER2 therapy efficacy and can serve as a predictor of treatment outcomes. More importantly, Bychkov and colleagues found that their CNN model could identify CISH HER2‐negative cases that may benefit from anti‐HER2 treatments. This finding challenges the conventional assumption that only patients with HER2+ status require anti‐HER2 therapy and suggests that there may be a broader population of patients who could benefit from this treatment.

The relationship between pCR and the tumor immune microenvironment in breast cancer has been the subject of frequent investigation, although not always in a systematic manner. For instance, a high Ki‐67 index (≥50%) has been identified as an independent predictive factor for pCR in patients with HER2‐positive BC [79]. Moreover, PD‐L1 expression correlated with tumor‐infiltrating lymphocytes (TILs) and emerged as a significant predictor of pCR [80]. In 2023, Huang et al. [81] utilized multistained histopathological images to develop an automated workflow for predicting pCR in BC from pre‐NAC biopsies. The researchers integrated IHC data on PD‐L1, CD8+ T cells, and CD163+ macrophages with HE‐stained images of the tumor immune microenvironment to create a composite feature set for predicting response to NAC. This machine learning model from WSI feature extraction pipeline demonstrated high accuracy in predicting the response to NAC in patients with HER2+ BC (AUC = 0.8975) and surpassed the performance of features manually generated by pathologists. This ML model, characterized by its automation, precision, comprehensiveness, interpretability, and reproducibility, highlights how signals from the tumor immune microenvironment can function as predictive markers or refine the selection of initial treatment. These insights could prove crucial in advancing anti‐HER2 precision oncology.

#### Predicting recurrence

As a highly heterogeneous cancer, approximately 20% of patients with HER2+ BC experience metastasis or recurrence after NAC or other treatments [82]. Personalized treatment options can be provided in advance by predicting the likelihood of postsurgical recurrence.

Yang et al. [60] proposed a novel CNN model using HE‐stained WSI features and clinical data to precisely predict recurrence risk in HER2+ cases. The ResNet50 model was used to extract image features and clinical data from enrolled samples. The samples were then split and validated using twofold cross‐validation. The results indicate that using computer‐aided systems based on HE images features can enhance predictive ability compared to clinical data alone.

In summary, AI technology combined with HE imaging has demonstrated potent applicability in predicting the efficacy of anti‐HER2 therapy owing to its numerous advantages, including higher accuracy, lower cost, faster speed, and less trauma.

### AI predicts anti‐HER2 therapy efficacy by imaging‐omics features

Aside from pathological imaging, various radiological imaging techniques were used to predict the efficacy of anti‐HER2 therapy. Each method has its unique advantages and disadvantages. Positron emission tomography [83] can be used to monitor tumor response to chemotherapy [84]. Its prominent shortcomings include requiring a radionuclide contrast agent and high cost. DCE‐MRI has also been used to forecast chemotherapy results in patients with BC [85]. Despite some promising outcomes, their high costs make them less accessible. Recently, diffuse optical imaging (DOI) has been explored for monitoring patient response to chemotherapy in BC [85]. Because of the lengthy process for acquiring data and reconstructing a volume image with reasonable resolution, DOI is not widely used as a standard method in clinical practice. Studies have demonstrated that quantitative ultrasound (QUS) methods can yield promising results in tissue characterization and tumor response monitoring [47]. It has also been used to assess BC response to chemotherapy [86]. Despite its potential, ultrasound imaging is unable to provide the same quality of 3D volumetric information as CT or MRI, restricting its widespread use.

Imaging histology, utilizing AI‐based characterization, offers more detailed information than human observation by identifying macroscopic, cellular, and molecular features. AI‐based predictive models can also furnish dependable and noninvasive biomarkers for assessing treatment response. Compared to core biopsy specimens, radiomic analysis provides a comprehensive overview of tumor pathological features by offering a noninvasive evaluation of the entire tumor. This led to the widespread application of AI‐based radiological imaging techniques.

#### AI‐based MRI imaging in anti‐HER2 therapy

In cases of HER2+ BC, MRI is the most accurate method for predicting pCR after NAC [87] among various quantitative imaging techniques. Furthermore, studies have demonstrated that DCE‐MRI is effective in evaluating tumor biological features, including subtypes [88], genomics [89], and receptor status. MRI provides additional predictive value beyond existing biological, clinical, and pathological predictors. This technique holds the potential to stratify patients and formulate personalized treatment plans.

Cain developed a multivariate machine learning model based on computer‐extracted features from pretreatment DCE‐MRI that could predict pCR for neoadjuvant therapy [61]. In the triple‐negative/HER2+ (TN/HER2+) subgroup, the AUC for predicting pCR was 0.707, indicating better predictive performance for these two subtypes, which are associated with poor prognosis.

Bitencourt et al. used MRI to evaluate HER2 gene amplification and pCR after NAC in HER2+ BC cases [62]. The machine‐learning model was developed using five‐fold cross‐validation and coarse decision trees. Radiomics and clinical MRI‐based parameters were compared using machine‐learning modeling, followed by correlation and ROC analyses. The optimal model for predicting HER2 heterogeneity achieved a diagnostic accuracy of 97.4%. The model that included both radiomic and clinical MRI parameters performed best for predicting pCR and had a diagnostic accuracy of 83.9%, sensitivity of 86.5%, and specificity of 80.0%. This machine learning model has the potential to help identify patients who would benefit from anti‐HER2 treatment and play a significant role in clinical practice.

Mazurowski and colleagues used computer vision algorithms to extract and select prognostic imaging characteristics from DCE‐MRI scans of patients diagnosed with invasive BC [17]. They found that 20 radiological features were significantly linked to distant RFS. Among these, heterogeneity, size, and perfusion had the highest predictive values with C‐indices of 0.64, 0.77, and 0.70, respectively.

Several studies reported that intratumoral HER2 pathological heterogeneity is associated with a less favorable prognosis [90]. In 2018, Wu et al. [63] also supported this standpoint. Using contrast‐enhanced MRI, researchers identified intratumoral subregions using network analyses. These subregions correspond to varying levels of perfusion and quantify their interactions. Following adjustments for clinical factors such as receptor status, pathological response, age, and tumor volume, a radiological signature was found to be predictive of RFS. This signature showed the distribution and abundance of poorly perfused regions. Breast MRI scans demonstrated a higher prevalence of poorly perfused regions in tumors associated with poor prognosis than in indolent tumors. Drukker and colleagues developed a DL model using long short‐term memory based on radiological features that correlate with the kinetics of dynamic MRI contrast enhancement throughout the course of NAC treatment. This model predicted the 2‐year RFS and achieved a C‐index of 0.80 [64]. This study used a recurrent neural network (RNN), a specialized DL architecture, to assimilate and learn from imaging features gathered across multiple time points.

Axillary lymph nodes (ALN) are the main route of metastasis in BC. According to a 10‐year randomized clinical trial [91], assessing ALN status plays a crucial role in early‐stage invasive cases. Currently, the standard methods for evaluating ALN status include sentinel lymph node biopsy (SLNB) and axillary lymph node dissection (ALND) surgery. However, these methods carry a risk of operation‐associated complications, such as decreased upper‐extremity range of motion, axillary web syndrome, lymphedema, and numbness. Yu et al. [65] developed an ALN‐tumor radiological signature for axillary lymph node status prediction using tumor and ALN radiological characteristics. They applied a machine‐learning method to identify predictive MRI features. The results indicated that the multiomics model, which integrates clinical and pathological characteristics, tumor MRI radiomics, lymph node MRI radiomics, and molecular subtypes, performed the best. Furthermore, significant differences were observed in predictive radiological features before and after NAC treatment, with a particular emphasis on the gray‐level dependence matrix features. Moreover, certain tumor microenvironment features are related to MRI radiomics, including types of methylated sites and long noncoding RNAs. These findings may explain the significant changes observed in key radiological features before and after NAC. This study demonstrated that a multiomics model can accurately predict ALN metastasis in early‐stage invasive BC. The more data realms included, the more accurate is the prediction. This model can guide clinical decision‐making by preventing unnecessary SLNB or ALND surgeries in patients with its high predictive ability.

HER2+ breast tumors have a higher ratio of multifocal/multicentric disease and skin‐nipple‐peri‐areolar involvement than in other molecular subtypes [92]. Image analysis combined with AI can assist radiologists in segmenting regional fields and quantifying imaging features. Several computational software programs have recently been developed for this purpose. Dashevsky and colleagues employed a leave‐one‐out cross‐validation‐based support vector machine (SVM) model to predict the resectability of HER2+ patients who underwent breast conservation surgery (BCS) [66]. Using 21 preoperative MRI‐contoured tumor features, the model achieved a sensitivity of 71.4% and a specificity of 74.4%. These results indicate that an MRI‐based AI model helps in surgical decision‐making by recommending wide margins or mastectomy for patients who might otherwise require re‐excision.

#### AI‐based QUS imaging in anti‐HER2 therapy

US imaging is commonly used to assess responses to NAC before, during, and after treatment. According to the China Anticancer Association breast cancer guidelines, patients should undergo US every two cycles after NAC therapy to assess treatment response [93]. US is more accessible, cost‐effective, and comfortable for patients compared to MRI. Jiang and colleagues hypothesized that a DL radiological model combining pre‐ and posttreatment US images could accurately predict pCR. Pretreatment images are associated with primary tumor characteristics and posttreatment images directly reflect response status [67]. To select features and construct radiomic signatures, researchers have utilized the minimum redundancy maximum relevance algorithm and least absolute shrinkage and selection operator for regression. A nomogram utilizing DL radiomics was created by leveraging radiomic signatures to independently detect clinicopathological risk factors. The evaluation of model performance encompassed calibration, discrimination, and clinical utility. The prediction within the HER2+ subgroup performed well, with an AUC of 0.95. Liu and colleagues devised and validated a Siamese Multi‐Task Network for the purpose of forecasting pCR based on longitudinal US images [68]. They enrolled 393 biopsy‐proven patients with HER2+ BC. The proposed Siamese multitask network consists of two subnetworks and integrated multiscale features from longitudinal US images captured both before and after NACT cycles, along with extracted dynamic information.

In summary, while single‐omics models have demonstrated satisfactory outcomes, their applicability is constrained to particular categories of factors, including pathological images, radiology, and clinical indices. Such models analyze disease prognosis only at the image or molecular level and do not fully utilize big data generated from other aspects. This may have omitted other factors that affect disease prognosis. Therefore, AI methods based on multiomics data have promising development prospects.

### AI assists in developing novel anti‐HER2 therapeutics

One of the key challenges in anti‐HER2 treatment is identifying of specific EGFR and HER2 inhibitors. Various computational approaches, including quantitative structure–activity relationships, virtual screening, and pharmacophore modeling, are used to identify potential inhibitors [94, 95, 96]. Previous classification models were developed to distinguish between inactive and active EGFR [97]. Before 2022, no machine learning model had the capability to selectively identify inhibitors targeting specific EGFR and HER2 isoforms. Therefore, there is an urgent need to develop a prediction system for guiding the design of isoform‐specific inhibitors. Saini and colleagues constructed a knowledge‐based computational model that accurately predicted the specificity of molecules for EGFR and HER2 isoforms. The model was based on a data set of 519 compounds that inhibit both EGFR isoforms [69]. They developed 72 classification models using 9 fingerprint types, 4 classifiers, and 2 different datasets (EGFR and HER2 isoforms). For HER2‐ and EGFR‐specific data sets, the models developed using IBK and random forest performed the best. In each data set, functional group and scaffold analyses were used to identify prevalent cores and fragments. They also developed an application called EGFRisopred by combining the most accurate models, which allows users to forecast the specificity of a compound for EGFR or HER2. By providing researchers with this free tool, new inhibitors of these targets can be identified.

To comprehend the structural implications of current trastuzumab mutations exhibiting elevated affinity values and effectively discern a high‐affinity mutant variant of trastuzumab targeting HER2, Balakrishnan et al. [70] developed a Support Vector Regression (SVR) model for predicting epistatic events in combination mutants. Researchers studied 193 single trastuzumab mutants, including their affinity values and various amino acid sequence‐derived descriptors. SVR hyperparameters and descriptor subset selection were achieved using a Genetic Algorithm‐Support Vector Regression (GA‐SVR) wrapper. The final machine learning model accurately predicted a set of six combination mutants and one single mutant, with an $R_{\mathrm{pre}}^{2}$ value of 0.72. According to the descriptor analysis in this model, variations in trastuzumab‐binding affinity are due to secondary structural changes induced by mutations. Trastuzumab wild‐type and mutant variants were subjected to duplicate molecular dynamics simulations of 100 and 20 ns, respectively. This study discovered that mutations in the Complementarity‐Determining Region 3 of the heavy chain (CDR‐H3) can accelerate the development of more effective trastuzumab with significantly better binding affinity and marketability.

### AI predicts anti‐HER2 therapy outcomes

Breast cancer brain metastasis (BCBM) is a common complication of HER2+ BC. However, a recent meta‐analysis of studies published between 2000 and 2020 found that BCBM incidence varied among different subtypes. The pooled cumulative incidence of CBM in patients with HER2+ BC was 31% [98]. Other studies have reported that 13% of patients with metastatic HER2+ BC develop BCBM annually. The prevalence of BCBM is 67% in patients with mBC and germline BRCA1/BRCA2 mutations [99]. Early symptoms of brain metastases are often mild and can be easily overlooked by patients. Symptoms such as headache, vomiting, and occasional loss of consciousness may not be recognized as brain metastasis. BCBM requires a different treatment approach than metastases to other sites. Current guidelines do not recommend routine monitoring of BCBM in patients with primary BC because of the difficulty in identifying those at higher risk [100]. Some patients may delay seeking medical attention until their symptoms become severe and unbearable, potentially missing an optimal window for treatment [101].

Takada and colleagues developed a mathematical model aimed at forecasting the risk of BCBM in individuals with HER2+ primary BC undergoing NAC and trastuzumab postsurgery [102]. The model predicted BCBM events, defined as either the brain being the first site of recurrence or the discovery of BCBM following metastatic treatment. Baseline clinicopathological factors were used to develop the model, which was trained using an alternating decision tree algorithm (ADTree) capable of quantitatively predicting outcomes. Model accuracy for identifying patients at high risk for BCBM patients was validated with an AUC of 0.871. The only flaw is it lacks an external cohort to validate the model, and stricter verification is needed in the future.

## 5 PROSPECTS FOR THE FUTURE: WHERE ARE WE HEADED?

AI is a novel tool in medicine that has the potential to predict treatment efficacy. Although most studies have shown that their models have met or even exceeded clinician performance, few have successful applications in the real world [103]. The reasons for the difficulty in implementation may include an insufficient standard database, unified industry standards, specific clinical application scenarios, policies, and regulatory support. Furthermore, with digitalization and increasingly widespread sharing of medical records in recent years, the problems of information leakage and privacy protection have gained prominence [104].

Currently, there are a limited number of prospective studies and randomized clinical trials examining AI in anti‐HER2 therapy, with most of them having a high degree of bias. Therefore, we should strengthen the correlation between experimental design and clinical reality as well as reduce systematic error. Previous studies and application prospects should also be evaluated rationally, patient interests should be protected, research waste should be reduced, and research findings should not be blindly exaggerated. The second challenge is the difficulty in reproducing AI models because of the limited availability of code and data sources. The stability of different models is difficult to assess because of various inconsistent preprocessing methods and prediction goals. Therefore, we should engage more centers with big data and encourage sharing codes and developing standardized datasets to support AI models across multiple disciplines. The third step is to integrate multiomics information to achieve a more accurate, individualized, and predictable medical body. AI has the capacity to process and integrate vast quantities of data in a manner beyond human capability. A comprehensive AI‐driven predictive model for Anti‐HER2 therapy should encompass all pertinent clinical data pertaining to patients and facilitate the entirety of the medical treatment process. Consequently, it is imperative to enhance the integrity and objectivity of information collection to enable the smooth sharing of large amounts of data across different centers. To introduce AI into the healthcare system, we must understand the trust and acceptability of patients, medical staff, and the public. Moreover, for AI technology to be successful and useful, it needs a large number of people involved in collecting medical data, while protecting the privacy of large populations [105]. Therefore, establishing a sound supervisory and management system to control actual risks and ensure the safety of this emerging system is important [106, 107]. More importantly, it is imperative to establish a comprehensive legal and ethical framework to enable the sharing of large datasets and facilitate the widespread utilization of AI systems across multiple regions and centers. Finally, with the rapid emergence of anti‐HER2 drugs and their proven efficacy, AI‐based anti‐HER2 treatment models require more data to improve [108, 109]. The implementation of immune checkpoint inhibitors in tumor immunotherapy has demonstrated substantial clinical advantages, indicating its potential as an effective treatment approach. The integration of artificial intelligence, an advanced technology, enables the personalized prediction of tumor immunotherapy outcomes through the automation of model construction, thereby facilitating individualized treatment approaches for patients [110].

In summary, due to the intricate nature of predicting the efficacy of anti‐HER2 therapy, there is a necessity for mutual learning between AI and human intelligence. Collaboration among scientific researchers, businesses, and healthcare professionals is essential to establish comprehensive databases and industry norms, overcome technical obstacles, facilitate the creation of AI‐supported systems capable of precisely identifying suitable candidates for anti‐HER2 therapy, accurately forecasting outcomes, advancing the adoption of AI‐assisted treatments, and securing the confidence of both physicians and patients.

## 6 CONCLUSION

Artificial intelligence, as an innovative technology, helps integrate multiple datasets. AI automates the prediction of anti‐HER2 therapy effects based on constructed models, thus realizing individualized treatment for HER2+ BC patients. However, there are several challenges. In the future, it is anticipated that AI‐assisted systems can simulate tumor biological behavior and drug treatment responses more accurately. This benefits a large proportion of patients with HER2+ BC and enhances both the efficiency and quality of medical care.

## AUTHOR CONTRIBUTIONS

**Xinpei Deng**: Data curation; writing—original draft; validation. **Yixuan Yan**: Data curation; writing—original draft. **Zekai Zhan**: Writing—original draft; visualization. **Jindong Xie**: Validation; supervision; visualization; writing—original draft. **Hailin Tang**: Validation; supervision. **Yutian Zou**: Conceptualization; writing—review and editing. **Jian Tu**: Conceptualization; writing—review and editing. **Peng Liu**: Conceptualization; writing—review and editing.

## CONFLICT OF INTEREST STATEMENT

The authors declare no conflict of interest.

## ETHICS STATEMENT

No animals or humans were involved in this study.

## Data Availability

No new data and scripts were used for this review. Supporting Information (graphical abstract, slides, videos, Chinese translated version, and update materials) may be found in the online DOI or iMeta Science http://www.imeta.science/imetaomics/.
